# An NIR-II Responsive Nanoplatform for Cancer Photothermal and Oxidative Stress Therapy

**DOI:** 10.3389/fbioe.2021.751757

**Published:** 2021-10-15

**Authors:** Bin Huang, Yuanpeng Huang, Han Han, Qiuyue Ge, Dongliang Yang, Yanling Hu, Meng Ding, Yanqing Su, Yanbin He, Jinjun Shao, Jianfeng Chu

**Affiliations:** ^1^ Academy of Integrative Medicine of Fujian University of Traditional Chinese Medicine, Fuzhou, China; ^2^ Xiamen Hospital of Traditional Chinese Medicine, Xiamen, China; ^3^ School of Physical and Mathematical Sciences, Nanjing Tech University (NanjingTech), Nanjing, China; ^4^ Nanjing Stomatological Hospital, Medical School of Nanjing University, Nanjing, China; ^5^ Department of Pharmacy, Xiamen Children’s Hospital, Xiamen, China; ^6^ Fujian Key Laboratory of Integrative Medicine on Geriatrics, Fujian University of Traditional Chinese Medicine, Fuzhou, China

**Keywords:** photothermal ablation therapy, chemodynamic therapy, phase change material, drug delivery, responsive nanomaterial

## Abstract

Chemodynamic therapy as an emerging therapeutic strategy has been implemented for oncotherapy. However, the reactive oxygen species can be counteracted by the exorbitant glutathione (GSH) produced by the tumor cells before exerting the antitumor effect. Herein, borneol (NB) serving as a monoterpenoid sensitizer, and copper sulfide (CuS NPs) as an NIR-II photothermal agent were loaded in a thermo-responsive vehicle (NB/CuS@PCM NPs). Under 1,060-nm laser irradiation, the hyperthermia produced by CuS NPs can be used for photothermal therapy and melt the phase change material for drug delivery. In the acidity microenvironment, the CuS NPs released from NB/CuS@PCM NPs could degrade to Cu^2+^, then Cu^2+^ was reduced to Cu^+^ during the depletion of GSH. As Fenton-like catalyst, the copper ion could convert hydrogen peroxide into hydroxyl radicals for chemodynamic therapy. Moreover, the NB originated from NB/CuS@PCM NPs could increase the intracellular ROS content to improve the treatment outcome of chemodynamic therapy. The animal experimental results indicated that the NB/CuS@PCM NPs could accumulate at the tumor site and exhibit an excellent antitumor effect. This work confirmed that the combination of oxidative stress–induced damage and photothermal therapy is a potential therapeutic strategy for cancer treatment.

## Introduction

Due to the hypoxic tumor microenvironment, tumor cells undergo hypoxic metabolism and inhabit in an elevated level of redox homeostasis circumstance (Yang et al., 2020). As an adaptive response, tumor cells will improve their antioxidant capacity, for example, the increased glutathione (GSH) levels in tumor cells further endow the tumor cells with anti-apoptosis and drug-resistant performance (Bansal and Simon, 2018; Liu Z. et al., 2020). Even worse, the exorbitant GSH in tumor cells as a reactive oxygen species (ROS) scavenger not only dramatically reduces the therapeutic efficacy of ROS-medicated therapy but also facilitates tumor metastasis, which makes cancer with high mortality and more difficult to be treated (McCarty et al., 2010; Lin X. et al., 2019; Liu M. D. et al., 2020; Zhu et al., 2021).

Borneol (NB) is a bicyclic monoterpenoid that can be extracted from a variety of Chinese herbal plants, such as valerian, lavender, and chamomile (Su et al., 2013). NB possesses analgesic, antibacterial, and anti-inflammatory effects, which has been approved by the Food and Drug Administration and widely used in the fields of cosmetics, food, and pharmaceuticals (Bhatia et al., 2008; Cherneva et al., 2012). NB, which serves as a chemosensitizer, can potentiate the therapeutic effect of anticancer drugs (such as cisplatin, paclitaxel, and doxorubicin) through cellular redox homeostasis interference by activating ROS-mediated oxidative damage (Horváthová et al., 2009; Su et al., 2013; Cao et al., 2020). Unfortunately, the overexpressed glutathione (GSH) in the malignant tumor generally attenuates the therapeutic effect of ROS-medicated therapy. Therefore, the synergistic therapy with tumor microenvironment remodeling property is critical for the eradication of the malignant tumor (Liang et al., 2020).

Recently, many chemodynamic agents based on variable metal ions (such as Mo^5+^/Mo^6+^, Mn^2+^/Mn^4+^, Fe^2+^/Fe^3+^, and Cu^+^/Cu^2+^) have been widely implemented in tumor microenvironment reconstruction (Liu et al., 2019; Ashok et al., 2020; Sun et al., 2020; Yang et al., 2020; Li Y. et al., 2021; Sun et al., 2021). Using this strategy, a satisfactory outcome is shown in the improvement of the treatment effect. For example, Yang et al. reported PtCu_3_ nanocages with horseradish peroxidase–like and GSH peroxidase–like catalytic activity, which could consume GSH for boosting the therapeutic effect of chemodynamic therapy (CDT)/sonodynamic therapy (Zhong et al., 2020; Yang H. et al., 2021). The Zhao group fabricated a hydrogen peroxide (H_2_O_2_)–responsive tin ferrite (SnFe_2_O_4_) nanoparticle with GSH peroxidase–like, Fenton-like, and catalase-like performance for GSH depletion and NIR-I photothermal-CDT (Feng et al., 2021). However, NIR-I light with limited tissue penetration depth and the maximum permissible exposure is 0.33 W cm^−2^, which is worse than NIR-II light (Shi et al., 2020; Dai et al., 2021; Wang et al., 2021; Zhang et al., 2021). To overcome this predicament, Sun et al. prepared copper sulfide (CuS) with NIR-II photothermal ability functionalized manganese dioxide nanoparticles for GSH elimination and NIR-II photothermal augmented CDT because Cu^2+^ can be reduced to Cu^+^ by the intratumoral GSH and then the H_2_O_2_ in the tumor can be catalyzed into the hydroxyl radical for CDT (Ma et al., 2019). Therefore, the ingenious design of nanomaterials with GSH consumption and intracellular oxidation homeostasis interference is critical for improving the therapeutic effect of ROS-medicated therapy.

**SCHEME 1:**
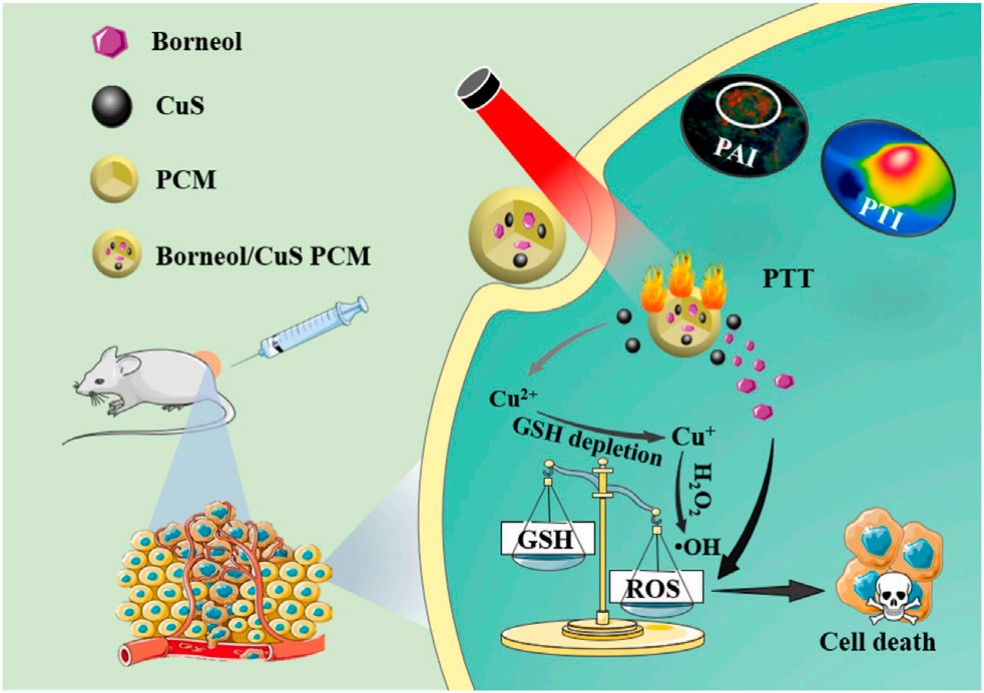
NB/CuS@PCM NPs for tumor photothermal and oxidative stress therapy.

Herein, the phase change material (PCM) was used to encapsulate CuS NPs and NB for disrupting intracellular redox homeostasis and thermal deactivation tumor cells. The NB serving as a chemosensitizer to enhance ROS generation and the CuS NPs working as NIR-II photothermal agent with GSH elimination and Fenton-like catalytic performance were encapsulated into the PCM vehicle to prepare the NB/CuS@PCM NPs for disturbing intracellular redox homeostasis after the PCM was melted by the heat produced by CuS NPs. Subsequently, H_2_O_2_ in the tumor cells was catalyzed by copper ions to generate hydroxyl radicals for CDT *via* Fenton-like reaction (Ma et al., 2019). Furthermore, the NB could improve intracellular ROS content to boost the CDT performance (Scheme 1). Combined with the oxidative stress–induced damage and photothermal therapy, NB/CuS@PCM NPs showed a pronounced antitumor effect *in vitro* and *in vivo*, demonstrating that NB/CuS@PCM NPs would be a potential candidate for oncotherapy.

## Materials and Methods

### Materials

Crude borneol was provided by Fujian Green Pine CO., Ltd. and further purified (purity ≥97%) by Fujian Nanping Green Pine Chemical Co., Ltd. Copper chloride (CuCl_2_), sodium sulfide, and gallic acid were offered by Sinopharm Chemical Reagent Co., Ltd. (Shanghai, China). Dichlorofluorescein diacetate (DCFH-DA), thiazolyl blue tetrazolium bromide (MTT), and propidium iodide (PI) were provided by Beyotime Biotechnology Co., Ltd., Shanghai, China). Glutathione, polyvinylpyrrolidone (K30), and 5,5′-dithiobis-(2-nitrobenzoic acid) (DTNB) were available from Shenggong Biotech Co., Ltd. (China). Murine mammary carcinoma 4T1 cells and the cell culture medium were obtained from Keygen Biotech Co., Ltd. (China).

### Synthesis of CuS NPs

100 mg of polyvinylpyrrolidone, 20 mg of gallic acid, and 86 mg of CuCl_2_ were dissolved in 10 ml of Milli-Q water. After stirring for 1 h, 200 mg of sodium sulfide was added. The solution color would change to black within seconds. After stirring for 4 h, the resulting product was dialyzed with a 10,000 Da-molecular weight cutoff dialysis bag and filtered using a 0.22-μm syringe filter.

### Preparation of NB/CuS@PCM NPs

The phase change material (PCM) consisting of 1-hexadecanol and oleic acid (3.5:1) was prepared as previously described (Ewulonu et al., 2019; Zhang et al., 2020; Yang D. et al., 2021; Xu et al., 2021). To construct the NB/CuS@PCM NPs, 20 mg of ovolecithin, 10 mg of DSPE-PEG, and 10 mg of CuS NPs were dissolved in 10 ml of water and warmed up to 50°C. The ethanol mixture of PCM (10 mg) and NB (10 mg) was quickly injected into the aforementioned solution under sonication conditions. Then the resulting solution was cooled in an ice bath. After purification by dialysis and filtration, NB/CuS@PCM NPs were harvested. The loaded content of NB in CuS/NB@PCM NPs was measured using a UV-vis spectrometer through the chromogenic reaction (Li et al., 2008).

### Characterization

The size distribution and morphology of NPs were analyzed *via* a dynamic light scatterer, scanning electron microscope, and transmission electron microscope. The powder X-ray diffraction (XRD) pattern was implemented *via* a D8 Advance X-ray diffractometer. A UV-3600 UV-vis-NIR spectrometer was used for the collection of absorbance spectra. An E50 infrared camera was adopted to measure the sample temperature change.

The release behavior of NB was monitored using vanillin as a chromogenic reagent, as in the method Li described previously (Li et al., 2008). In brief, a certain amount of the concentrated sulfuric acid solution containing 10 mg ml^−1^ vanillin was added to 250 μL of the sample or NB standard solution. The resulting solution was placed at room temperature for 10 min. Finally, the resulting solution was diluted (1:1) with water before monitoring the absorbance spectrum change.

### Photothermal Measurement

Different concentrations (0, 25, 50, and 100 μg ml^−1^) of NB/CuS@PCM NPs were exposed to a 1,060-nm laser (1 W cm^−2^). And the temperature variation of the sample was determined using a FLIR infrared camera. To investigate the effect of laser power density, NB/CuS@PCM NPs (100 μg ml^−1^) were exposed to the 1,060-nm laser (0.4, 0.6, 0.8, and 1 W cm^−2^). To evaluate the photothermal stability, the temperature variation of NB/CuS@PCM NPs (100 μg ml^−1^) was monitored during five laser (1 W cm^−2^) on–off cycles.

### GSH Depletion

The depletion of GSH was performed as our work previously described (Liu et al., 2019; Shi et al., 2020). In brief, different concentrations of NB/CuS@PCM NP solution (pH = 6.5) were mixed with GSH and DTNB probe (solvent, dimethyl sulfoxide/water, 1:1). The concentrations of GSH and DTNB probe were 10 and 10 mM, respectively. Then the absorbance spectra of samples were collected by using a UV-vis-NIR spectrometer.

### Chemodynamic Activity Assay

The methylene blue (MB) probe can react with the hydroxyl radical and then the blue color disappeared, which can confirm the emergence of the hydroxyl radical (Li Q. et al., 2021). To verify the catalytic performance of NB/CuS@PCM NPs, the NB/CuS@PCM NP solution treated with the 1,060-nm laser (1 mg ml^−1^) was incubated with different solutions (pH = 6.5), for example, NB/CuS@PCM NPs + MB, NB/CuS@PCM NPs + MB + H_2_O_2_. The concentration of MB and H_2_O_2_ was 10 μg ml^−1^ and 10 mM, respectively. After incubation for 3 h, the samples were characterized with a UV-vis spectrometer.

### Cell Viability Test

The MTT assay was used to evaluate the cytotoxicity of NB/CuS@PCM NPs (Yang et al., 2016; Cheng et al., 2021; Menaga et al., 2021; Yu et al., 2021). First, 150 μL of 4T1 cells (5 × 10^5^ cells ml^−1^) were added to each well (96-wells plate) and incubated for 16 h. Then the cells were treated with NB/CuS@PCM NPs and CuS@PCM NPs. To assess the photothermal therapeutic performance of nanoparticles, the cells were irradiated with the 1,060-nm laser. To assess the dark toxicity of nanoparticles, the cells were cultured without laser treatment. After cultivation for another 12 h, a routine cytotoxicity assay was implemented. Besides, after the cells were treated with NPs (100 μg ml^−1^) and laser, the photothermal therapeutic efficiency was also confirmed by calcein AM and PI fluorescent staining.

### Intracellular ROS Detection

The intracellular ROS generation was analyzed using a reactive oxygen fluorescent probe (DCFH-DA). First, NB/CuS@PCM and CuS@PCM NPs were treated with the 1,060-nm laser. And the resulting dispersion solutions (pH = 6.5, H_2_O_2_ = 50 μM) were co-cultivated with 4T1 cells for 1 day. Then the cell was stained with the DCFH-DA probe before fluorescent images were recorded using an X71 inverted fluorescence microscope (Olympus, Japan).

### *In vivo* Antitumor Assay

All animal studies were carried out according to the ethical principles of Guide For the Care and Use of Laboratory Animals of Nanjing Tech University. To probe the therapeutic effect of NB/CuS@PCM NPs *in vivo*, BALB/c mice (female, 4–5 weeks) bearing 4T1 cell xenografts were stochastically assigned to four groups and received different treatments: 1) PBS, 2) NB/CuS@PCM NPs (2 mg ml^−1^, 100 µL), 3) NB/CuS@PCM NPs (2 mg ml^−1^, 100 µL) + laser. Four hours after intravenous injection, the tumor was irradiated with the 1,060-nm laser. Afterward, the tumor size was determined using a vernier caliper, and the body weights were monitored simultaneously. After treatment for 14 days, the mice were euthanized and then the organs were collected, fixed in 4% formaldehyde, and embedded in paraffin for histopathological analysis.

## Results and Discussion

First, the CuS NPs were prepared according to the previously reported literature with minor modification (Yang et al., 2014). The sodium sulfide solution was added to the CuCl_2_ solution supplement with gallic acid to form the CuS NPs. The TEM image in Supplementary Figure S1A revealed that the diameter of CuS NPs was about 36 nm, which was consistent with the DLS result (Supplementary Figure S1B). As depicted in Supplementary Figure S1C, the XRD pattern of CuS NPs was consistent with the standard card (PDF#06–0,464). And the peaks located at 27.4, 29.2, 31.7, 48.0, 52.5, and 59.3° can be ascribed to the lattice planes of (101), (102), (103), (110), (108), and (116), respectively (Shen et al., 2019). The absorbance spectrum of CuS NPs exhibited that gallic acid modified CuS NPs possess a strong NIR-II absorbance (Supplementary Figure S1D). These results confirmed that the CuS NPs were successfully synthesized.

**FIGURE 1:**
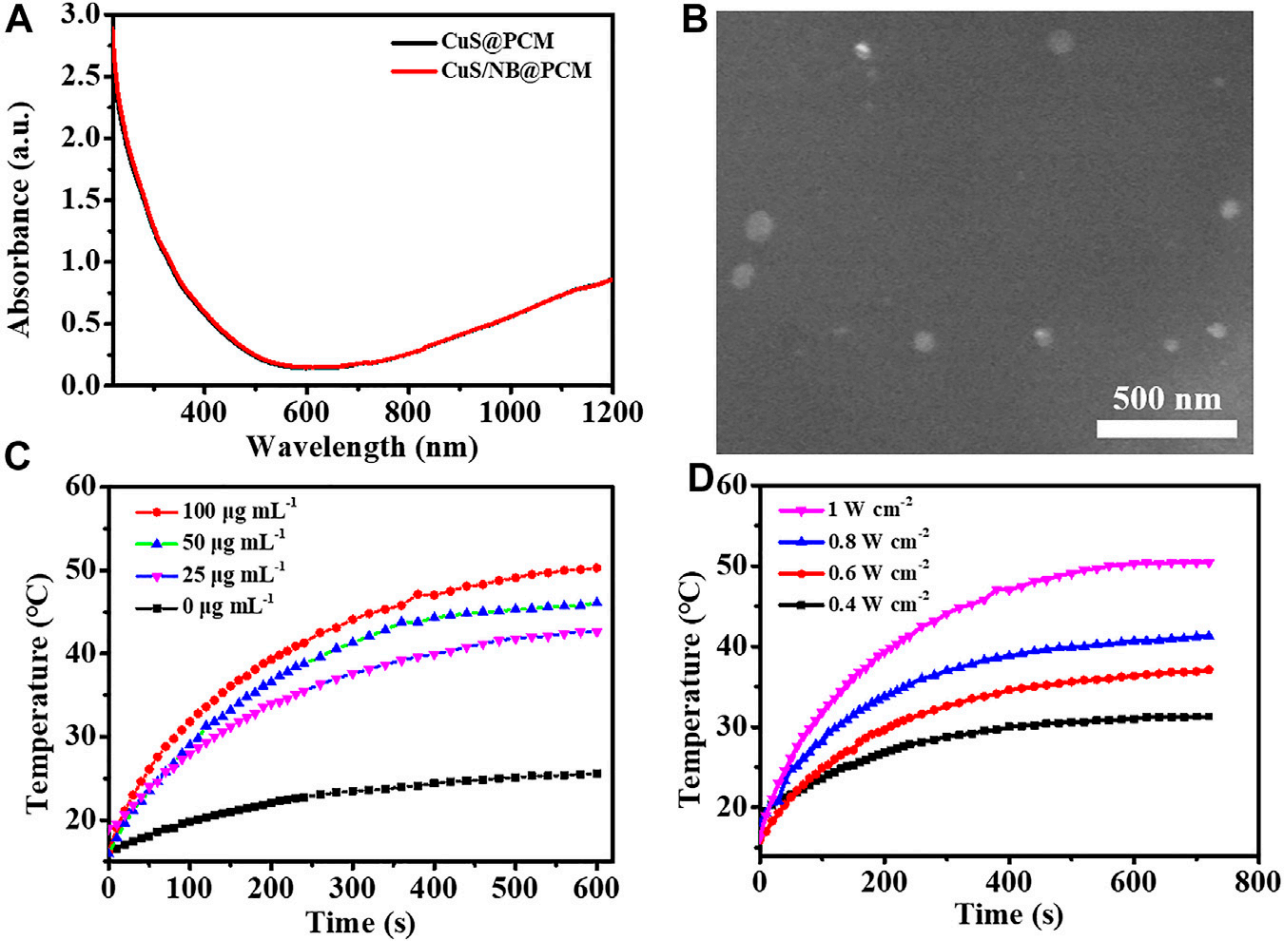
**(A)** Absorption spectra of CuS@PCM NPs and NB/CuS@PCM NPs. **(B)** TEM image of NB/CuS@PCM NPs. **(C)** Elevated temperature profile of different concentrations of NB/CuS@PCM NPs under 1,064-nm laser (1 W cm^−2^) exposure. **(D)** The elevated temperature profile of NB/CuS@PCM NPs (100 μg ml^−1^) under different densities of 1,064-nm laser exposure.

**FIGURE 2:**
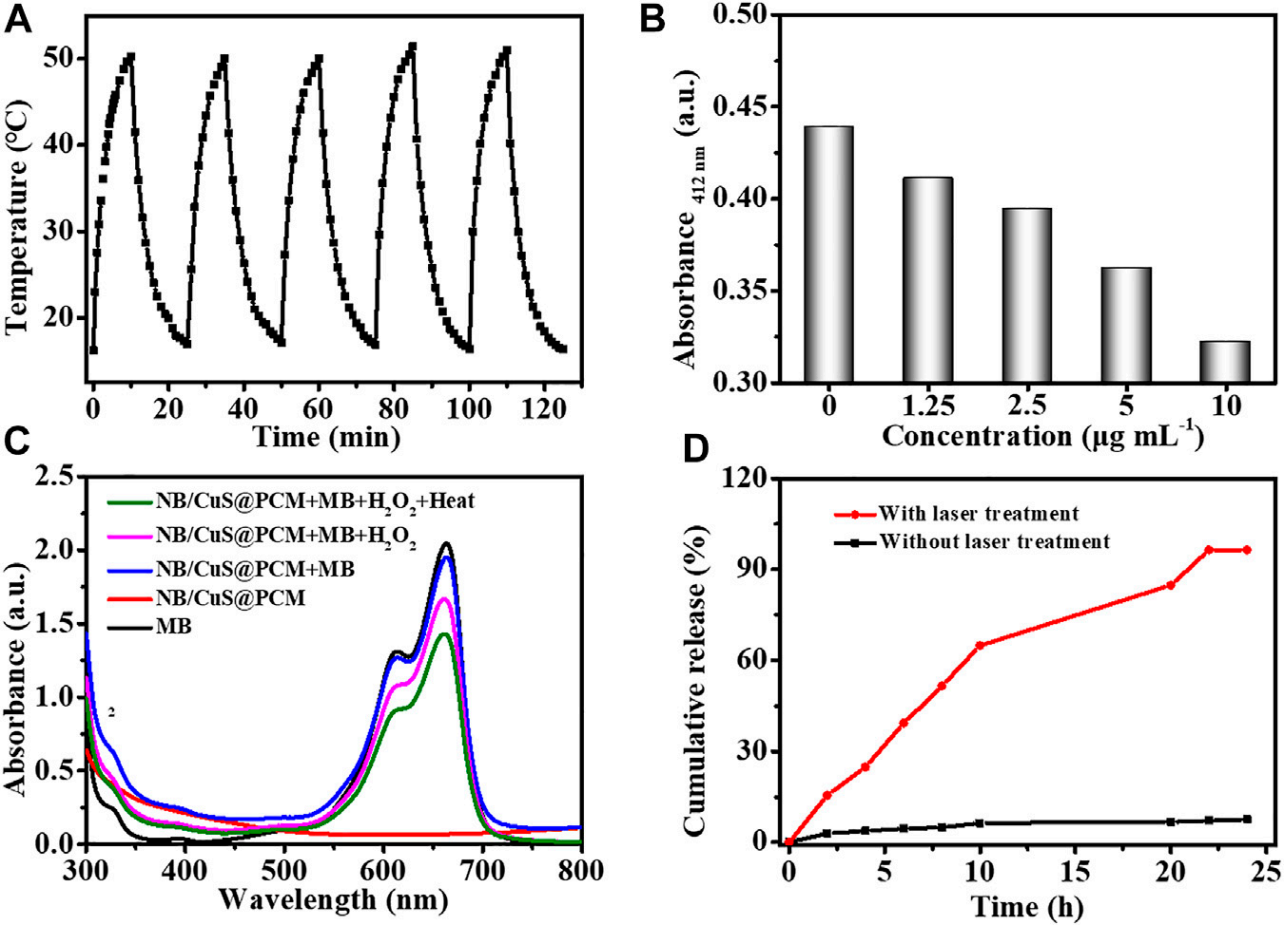
**(A)** Photothermal stability assay of NB/CuS@PCM NPs. **(B)** GSH-depleting performance of NB/CuS@PCM NPs. **(C)** Generation ability of the hydroxyl radical under different conditions. **(D)** NB release behavior under different conditions.

NB is a hydrophobic medical molecule, which sublimates easily and can be rapidly metabolized (Zhang et al., 2008). To realize the controllable delivery of NB and increase its medical effectiveness, NB was loaded to different vehicles (Cao et al., 2020; Li et al., 2015; Tang et al., 2015). To fabricate the on-demand therapeutic platform, the PCM carrier with temperature responsiveness was further used to load NB and NIR-II photothermal agent CuS NPs. The PCM consisting of 1-hexadecanol and oleic acid (mass ratio = 3.5: 1) has a suitable melting point of about 46°C (Zhang et al., 2020). After the PCM containing NB was dropped into the CuS NPs suspension solution, the NB/CuS@PCM NPs could be prepared when the solution was cooled in the ice water bath. As presented in Figure 1A, the absorption spectrum of NB/CuS@PCM NPs had no significant difference compare with CuS@PCM NPs. The SEM image indicated that the NB/CuS@PCM NPs held a spherical morphology with a mean diameter of 52.9 nm (Figure 1B, Supplementary Figure S2A). After treated with the 1,060-nm laser, the mean diameter of NB/CuS@PCM NPs decreased from 52.9 to 47.3 nm because PCM was melted by the heat produced by the CuS NPs (Supplementary Figure S2B). The loading efficiency of NB was measured to be 6.28%. Previous studies confirmed that the CuS NPs, which serves as a photothermal agent, have been widely used in biomedical application owing to their high photothermal conversion efficiency and high extinction coefficients in the NIR region (Yang N. et al., 2021). Then the photothermal performance of the NB/CuS@PCM NPs was confirmed under 1,060-nm laser illumination. As exhibited in Figures 1C,D, the temperature change of the NB/CuS@PCM NP dispersion revealed a dose and laser power dependence. In detail, the temperature of CuS/NB@PCM NPs (50 μg ml^−1^) could increase to 42.6°C after 1,060-nm laser irradiation (1 W cm^−2^) for 10 min. The eventual temperature of NB/CuS@PCM NPs (100 μg ml^−1^) increased from 31.3°C to 50.4°C as the laser power density increases from 0.4 W cm^−2^ to 1 W cm^−2^. The photothermal stability analysis indicated that the NB/CuS@PCM NPs possessed high stability even after four cycles of laser irradiation (Figure 2A). These results indicated that the CuS NPs encapsulated in the NB/CuS@PCM NPs could produce hyperthermia to melt the thermal-sensitive delivery system.

As we know, there is an acidic environment at the site of solid tumor (Lv et al., 2020). Under the NIR treatment, the CuS NPs released from the PCM were degraded to Cu^2+^. Then the Cu^2+^ can be transformed into Cu^+^ for Fenton-like chemodynamic therapy (CDT) under the reduction of the exorbitant GSH in the tumor (Wang et al., 2020). As shown in Figure 2B, in the acidic environments, the NB/CuS@PCM NPs could deplete GSH in a dose-dependent relationship. The hydroxyl radical generation ability of the NB/CuS@PCM NPs was performed under different conditions (Lin L.-S. et al., 2019). As displayed in Figure 2C, under heat conditions, the solution mixed with NB/CuS@PCM NPs, MB, and H_2_O_2_ exhibited weak absorbance intensity at 665 nm, confirming that heat can promote the generation of hydroxyl radicals. Next, the photo-activated drug release behavior of the NB/CuS@PCM NPs was investigated under 1,060-nm laser irradiation (Supplementary Figure S3). As exhibited in Figure 2D, under laser irradiation, a plentiful supply of NB was released, demonstrating the NB/CuS@PCM NPs could achieve on-demand NB release. In contrast, without NIR treatment, a small amount of NB was released from NB/CuS@PCM NPs. More importantly, the uptake of NB/CuS@PCM NPs was enhanced upon exposure to the 1,060-nm laser (Supplementary Figure S4).

**FIGURE 3:**
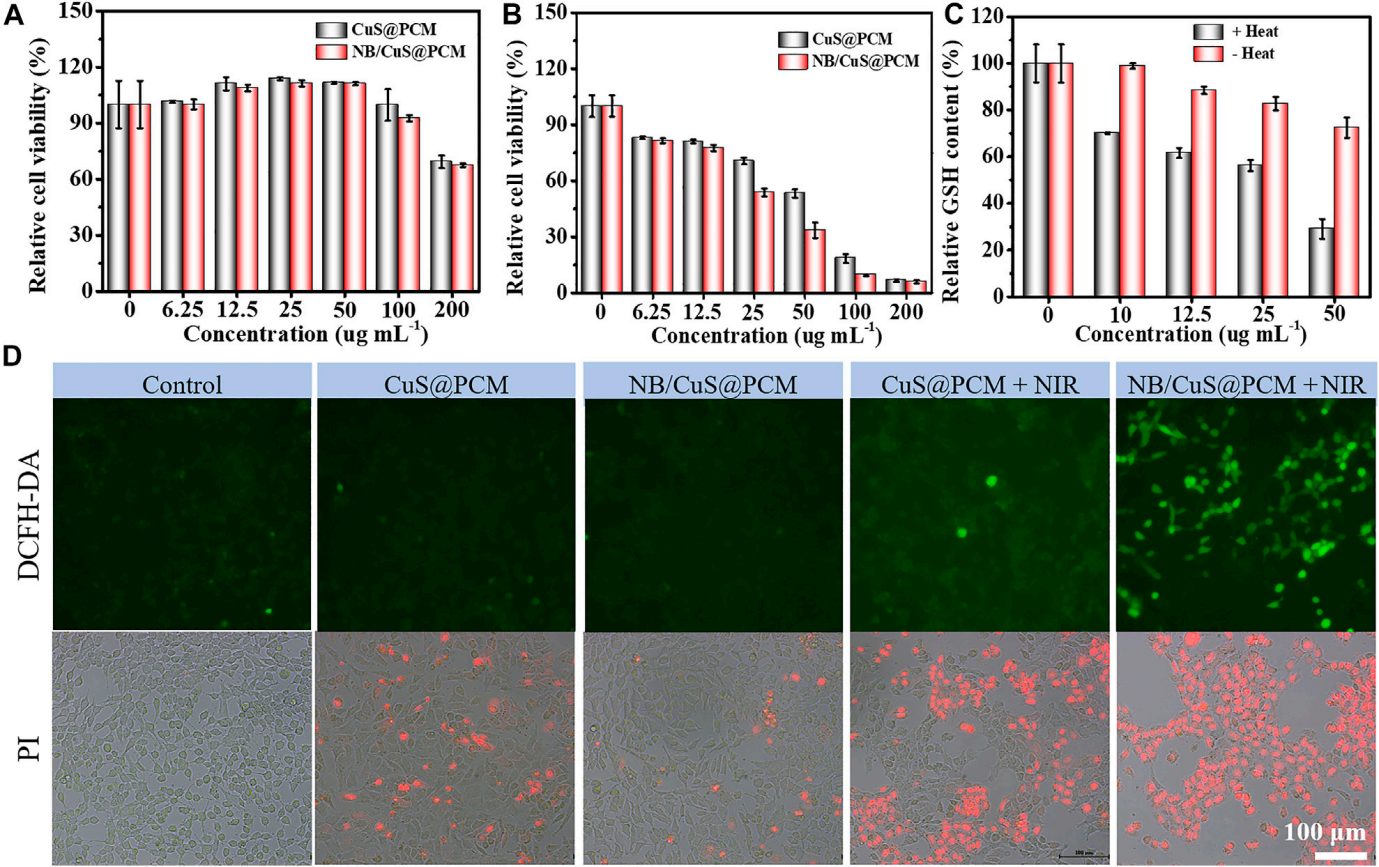
**(A)** Cytocompatibility assay. **(B)** Cytotoxicity assay of NPs under laser exposure. **(C)** Intracellular GSH-depleting capability of NB/CuS@PCM NPs. **(D)** Intracellular ROS content assay using DCFH-DA probe (green fluorescence). Cell death staining analysis using PI probe (red fluorescence).

After validating the catalytic and photothermal properties of NB/CuS@PCM NPs, the antitumor activities of NB/CuS@PCM NPs were evaluated *in vitro*. First of all, the cytotoxicity of NB/CuS@PCM NPs toward 4T1 cells was carried out. As shown in Figure 3A, in the dark environment, the cell viability of 4T1 cells was greater than 78% even NB/CuS@PCM NPs was concentration up to 100 μg ml^−1^. Upon exposure to 1,060-nm laser and NPs at 50 μg ml^−1^, the cell viability decreased sharply to 53.1% (for CuS@PCM) and 33.4% (for NB/CuS@PCM) (Figure 3B). Previous results indicated NB can improve the uptake of anticancer drugs and enhance its therapeutic effect by the increment of ROS content (Cao et al., 2020; Horváthová et al., 2009). The cells treated with NB/CuS@PCM NPs and laser produced a large amount of ROS (Figure 3D, DCFH-DA staining, Supplementary Figure S5). Moreover, the GSH content in the cells treated with NB/CuS@PCM NPs reduced significantly (Figure 3C). These results confirmed that the NB can enhance the therapeutic effect of CDT by the cellular redox homeostasis interference because of the NB with good biocompatibility when the concentration is lesser than 500 μg ml^−1^ (Supplementary Figure S6). Finally, the antitumor effect of NB/CuS@PCM NPs is also verified by PI staining (Yan et al., 2021). And the result was consistent with the MTT assay (Figure 3D, PI staining).

**FIGURE 4:**
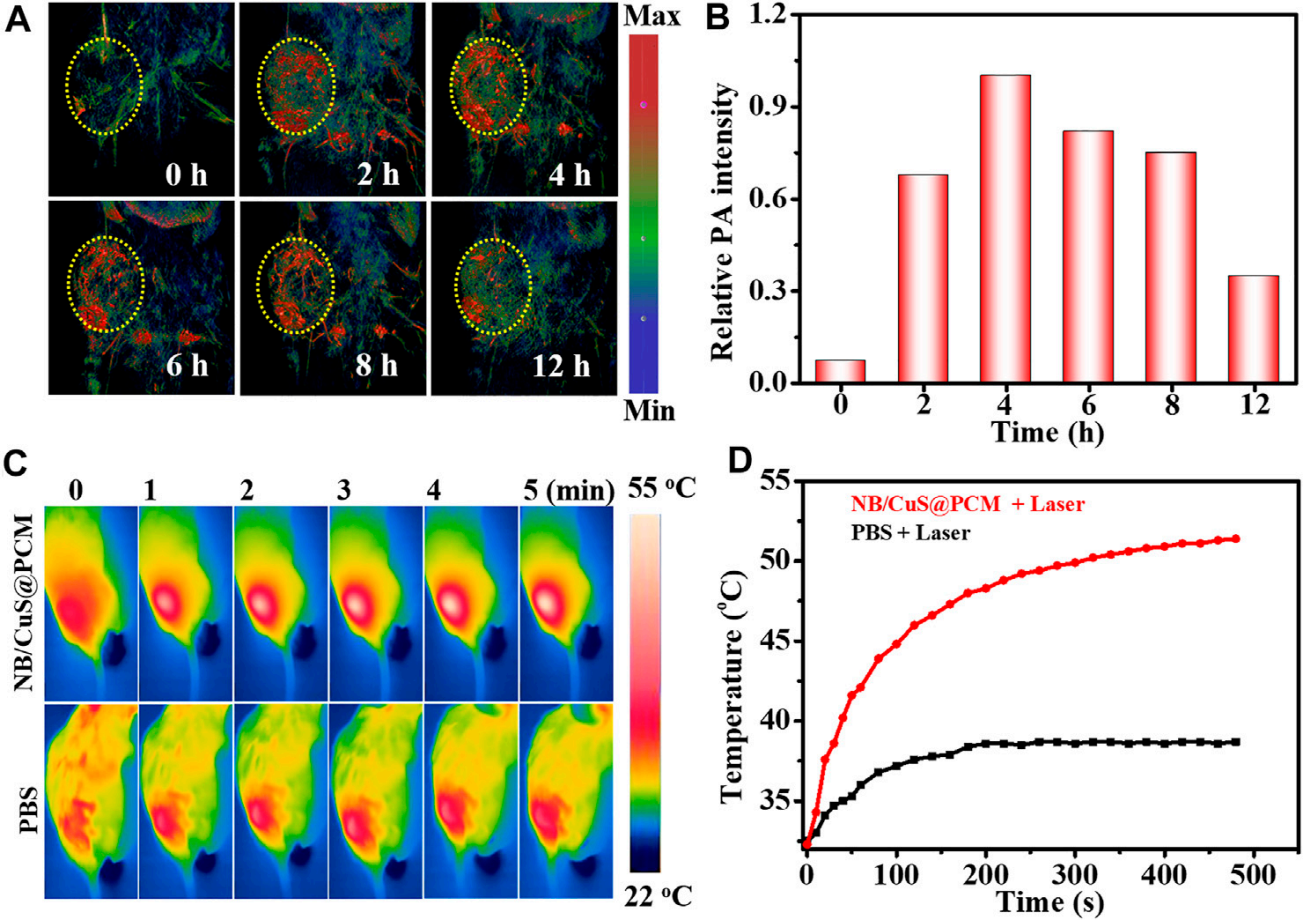
**(A,C)** NIR-II photoacoustic imaging photo and thermal imaging photo of tumor after the intravenous injection of NB/CuS@PCM NPs. **(B,D)** Photoacoustic signal and temperature change profile after the administration of NB/CuS@PCM NPs.

**FIGURE 5:**
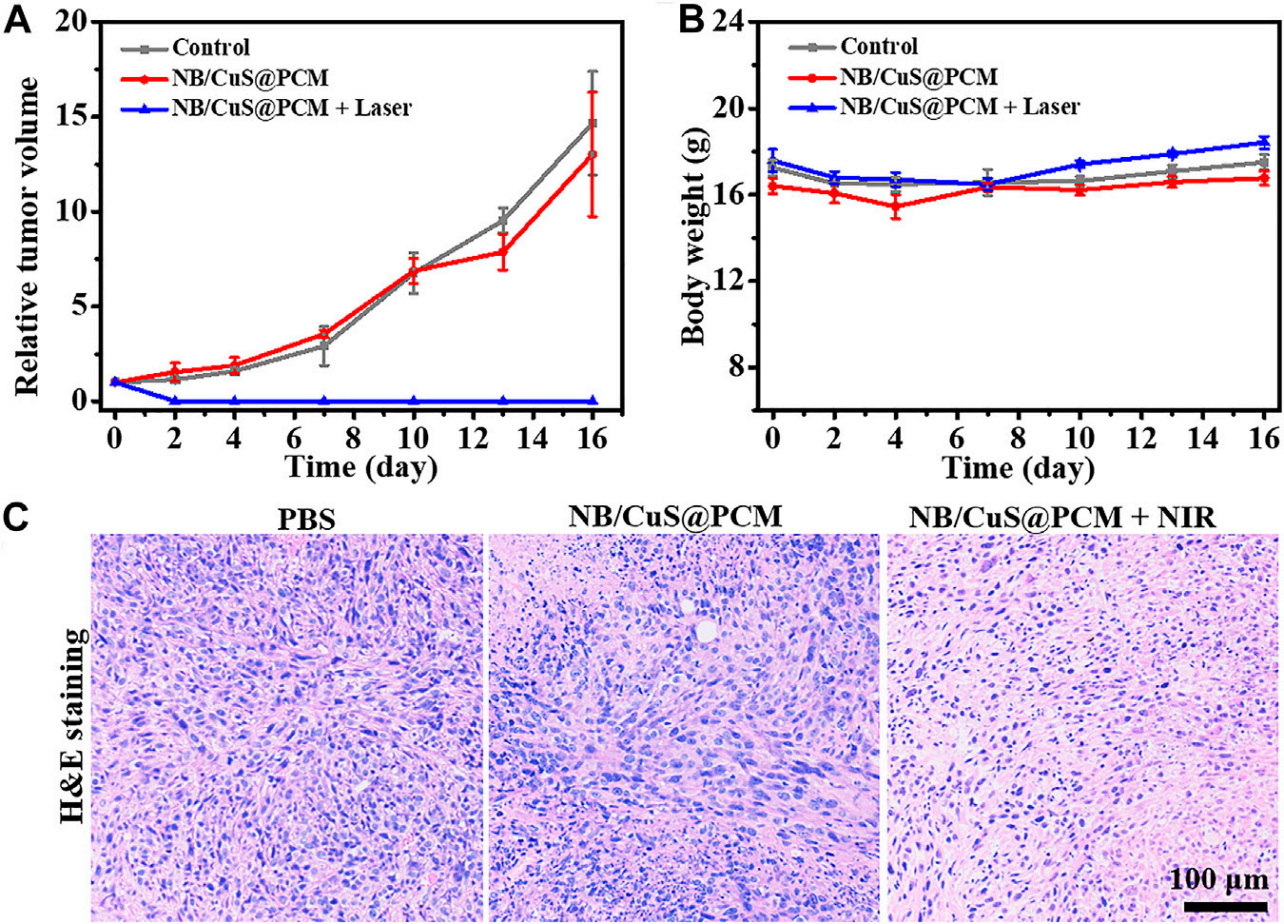
**(A)** Change of tumor volume after intravenous injection of NPs or PBS with and without NIR light exposure. **(B)** Weight fluctuation of mice during the treatment. **(C)** Histopathological analysis of tumor tissue by H&E staining after 1 day of treatment.

To further confirm the feasibility of tumor therapy *in vivo*, the photoacoustic imaging (PAI) performance and antitumor effect of NB/CuS@PCM NPs were investigated by using 4T1 tumor-bearing BALB/c mice. After intravenous medication, the accumulation of NB/CuS@PCM NPs in the tumor was monitored. As shown in Figures 4A,B, the photoacoustic signal intensity of the tumor reached a maximum 4 h after injection . Thus, 4 h after injection was chosen for photo-triggered drug release and photothermal therapy. Previous studies confirmed that the tumor produces exorbitant GSH and H_2_O_2_ (Yang et al., 2020). After the PCM was melted, the Cu^2+^ release from the CuS NPs in the acidic tumor environment was reduced to Cu^+^ by the exorbitant GSH (Chen et al., 2019). Thereafter, the Cu^+^ can react with the intratumoral H_2_O_2_ to produce a highly poisonous hydroxyl radical (·OH) by the Fenton-like process (Wang et al., 2020). As expected, in the NB/CuS@PCM NPs + laser group, the temperature at the tumor site raised rapidly and increased from 32.5 to 52.0°C in 5 min, which confirmed that the hyperthermally produced NB/CuS@PCM NPs were feasible to ablate the tumor cells and trigger the NB and CuS NPs release for enhanced chemodynamic therapy (Figures 4C,D). On the contrary, in the control group, the temperature elevated insignificantly, indicating that there was not a photothermal effect when the mice were only treated with the 1,060-nm laser. During treatment, the growth of the tumor was traced. As displayed in Figure 5A, in the groups treated with the 1,060-nm laser or NB/CuS@PCM NPs, the growth of tumor was not inhibited. After some time, the tumor volume increased 14.6-fold (for laser treatment) and 13-fold (for NB/CuS@PCM NPs treatment) after 16 days of treatment, while the tumor receiving NB/CuS@PCM NPs and 1,060 nm laser treatment was eliminated (Figure 5A; Supplementary Figure S7), which fully confirmed the antitumor effect of the NB/CuS@PCM NPs.

**FIGURE 6:**
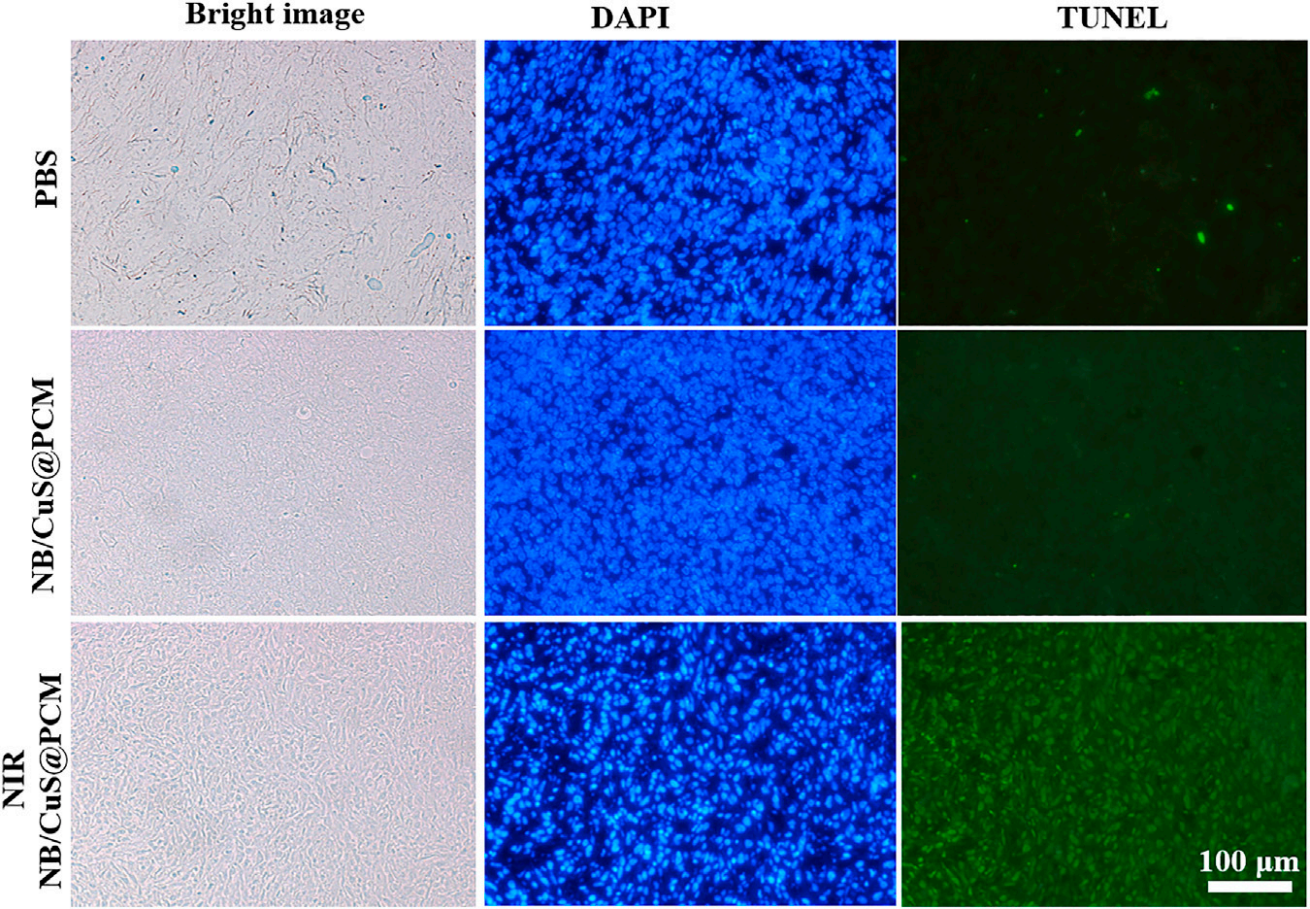
TUNEL assay of tumor tissue after 1 day of treatment. Nucleus was labeled by DAPI with blue fluorescence. The DNA nick was labeled by FITC modified dUTP with green fluorescence.

To evaluate the antitumor effect of NB/CuS@PCM NPs, the tumors were extracted for histopathological analysis. As shown in Figure 5C, an insignificant change was observed in the control group or the NB/CuS@PCM NP treatment group. On the contrary, in the treatment group of NB/CuS@PCM NPs + laser, a remarkable deformation could be seen, which indicated that the tumor cells might undergo apoptosis or necrosis, confirming that the NB/CuS@PCM NPs possess a strong antitumor effect with the assistance of laser (Figure 5C). To observe apoptotic cells in tissue sections, the terminal transferase–mediated dUTP nick end-labeling (TUNEL) was carried out (Li et al., 2017; Wu et al., 2018; Yang et al., 2019). As expected, after the tumor was treated with NB/CuS@PCM NPs and laser, substantial tumor cells were labeled with green fluorescence (Figure 6). This result was consistent with HE staining results.

Finally, the potential biotoxicity of the NB/CuS@PCM NPs was assessed by body weight change analysis, blood routine examination, and pathomorphology analysis. During the treatment, the body weight of the three groups had insignificant change, implying that the NB/CuS@PCM NPs possessed low physiological toxicity. In blood routine examination, various indicators in the blood, such as hemoglobin (HGB), red blood cells (RBC), white blood cells (WBC), and so on, exhibited no obvious fluctuations after the mice received treatment (Supplementary Figure S8). Besides, the major organs of mice were collected for histopathological analysis when the treatment was complete. As presented in Supplementary Figure S9, there were no prominent morphological changes, indicating NB/CuS@PCM NPs had excellent biocompatibility.

## Conclusion

In summary, we fabricated an NIR-II–activated nanoplatform (NB/CuS@PCM NPs) for photothermal therapy and intracellular oxidation homeostasis disturbance. The PCM could be melted by the hyperthermia under 1,060-nm laser irradiation to realize the thermo-responsive release of NB and CuS NPs. Under the acidic tumor environment, the copper ion dissociated from the CuS NPs was reduced into Cu^+^ *via* the consumption of GSH. Furthermore, intratumoral H_2_O_2_ was converted into highly poisonous ⋅OH to disrupt the intracellular oxidation hemostasis *via* Fenton-like reaction. Besides, the NB could increase the intracellular content of ROS for enhancing the therapeutic effect of chemodynamic therapy. *In vivo*, with the assistance of photoacoustic imaging, NB/CuS@PCM NPs exhibited a satisfactory therapeutic effect in cancer therapy.

## Data Availability

The original contributions presented in the study are included in the article/Supplementary Material; further inquiries can be directed to the corresponding authors.
