# Supplementary material for: An NIR-II Responsive Nanoplatform for Cancer Photothermal and Oxidative Stress Therapy
Source: Front Bioeng Biotechnol. 2021 Oct 15;9:751757. doi: 10.3389/fbioe.2021.751757 (PMC8553991; doi:10.3389/fbioe.2021.751757)
Supplement: Supplementary file 1 [file DataSheet1.docx]

Supplementary Material

## Supplementary Figures


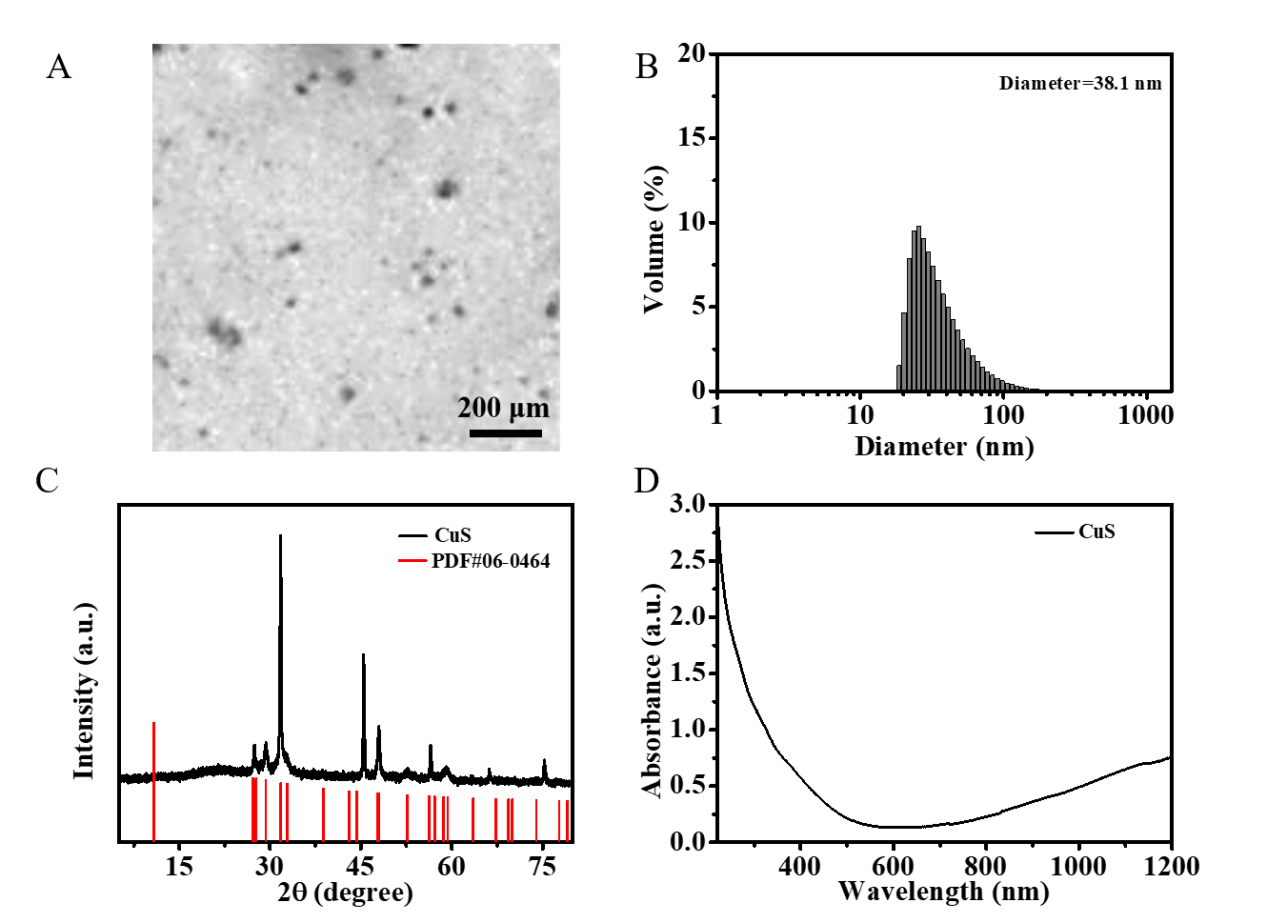


**Supplementary Figure 1.** (A) TEM image of CuS NPs. (B) Size distribution of CuS NPs. (C) XRD pattern of CuS NPs. (D) Absorption spectrum of CuS NPs.


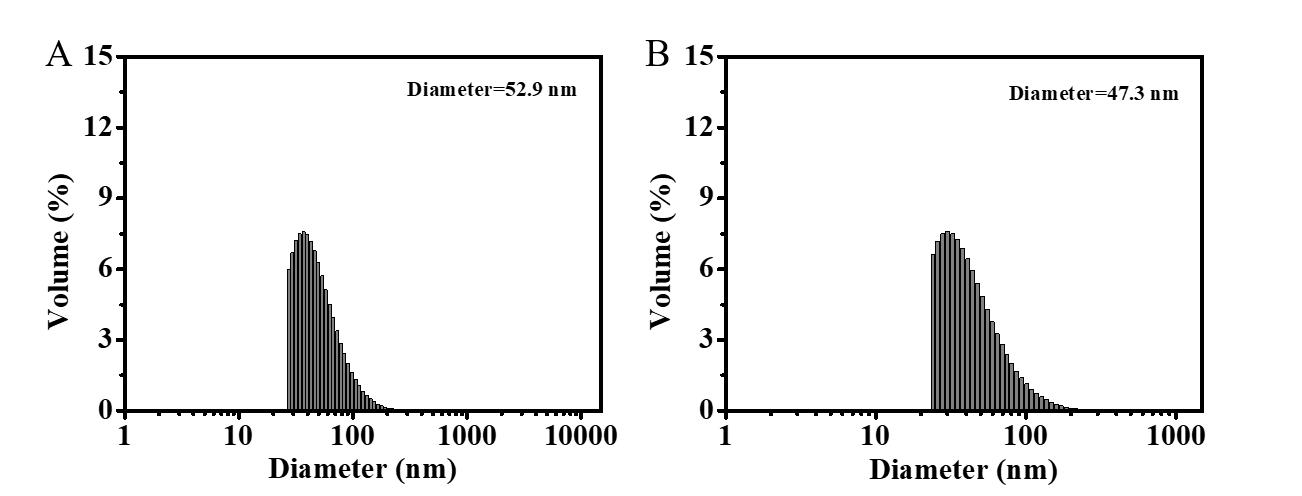


**Supplementary Figure 2.** Size distribution of NB/CuS PCM NPs before and after laser treatment.


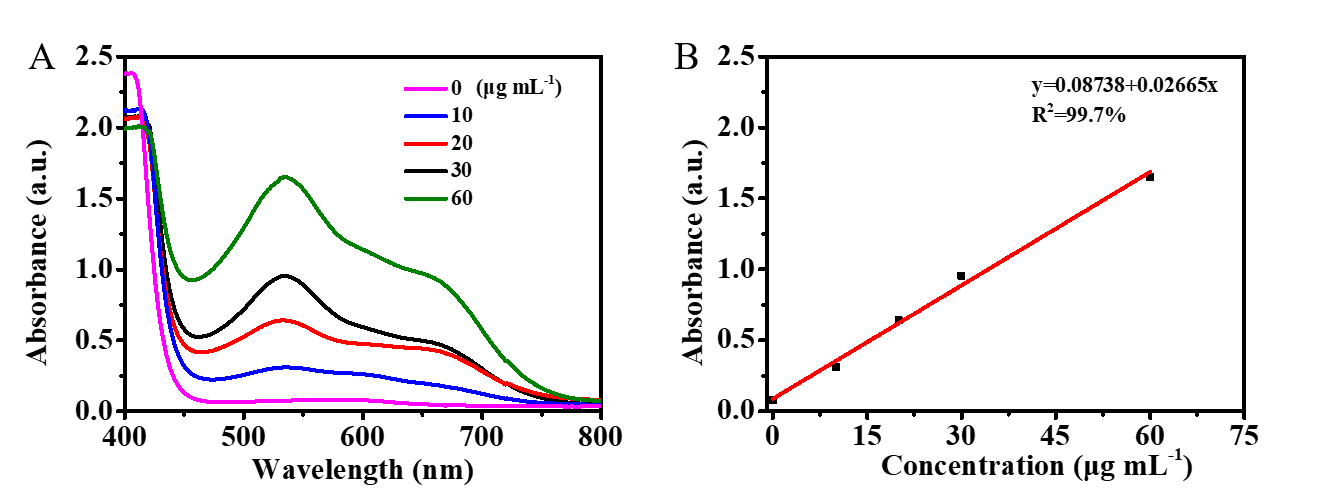


**Supplementary Figure 3.** (A) Absorption spectra change of NB and vanillin in the concentrated sulfuric acid solution. (b) The plot of absorbance at 535 nm versus the concentration of NB.

**Supplementary Figure 4.** The change in cellular NB/CuS PCM NPs intake.

**Supplementary Figure 5.** The change of intracellular ROS content after different treatments.

**Supplementary Figure 6.** The cytotoxicity of NB towards to 4T1 tumor cells.


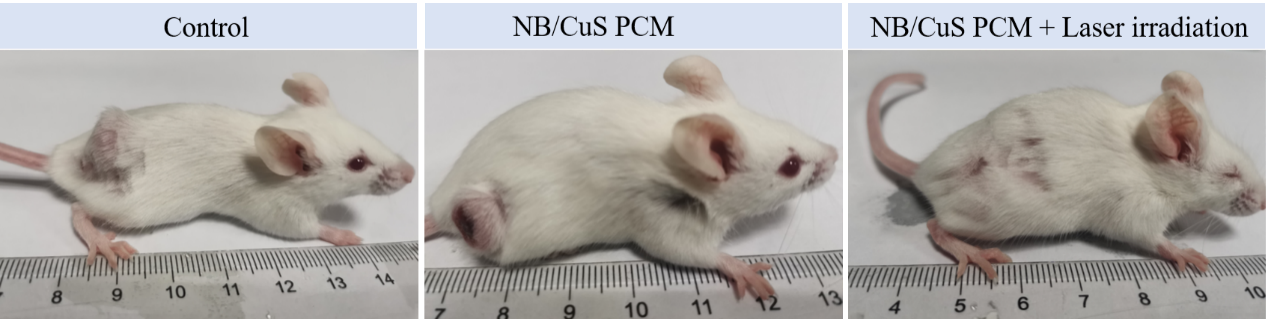


**Supplementary Figure 7.** Representative tumor images were recorded at the end of treatment.


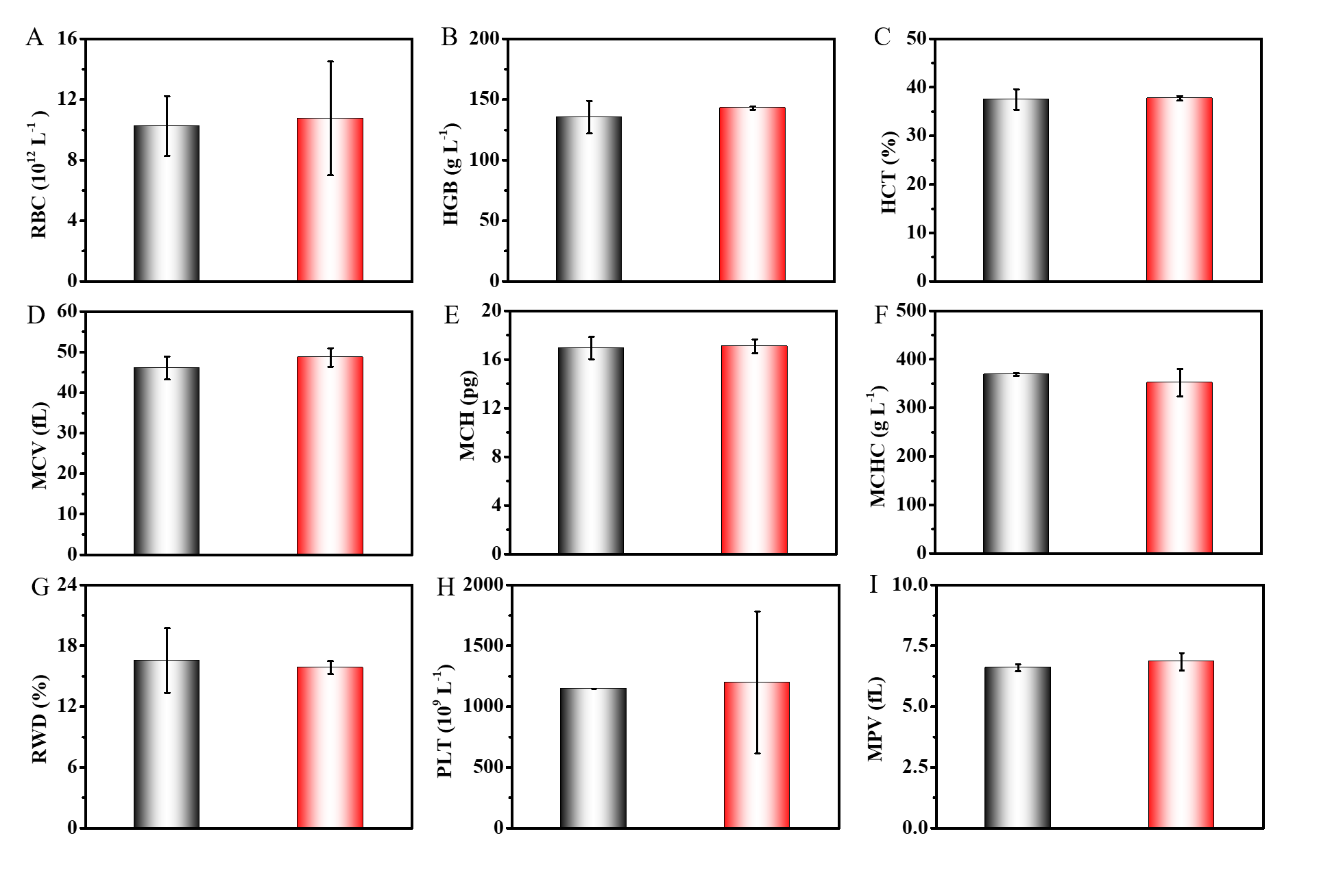


**Supplementary Figure 8.** Blood routine test at the end of treatment. The black and red columns are control and NB/CuS PCM NPs treated groups, respectively. RBC: red blood cell, HGB: hemoglobin, HCT: haematocrit, MCV: mean corpuscular volume, MCH: mean corpuscular haemoglobin, MCHC: mean corpuscular haemoglobin concentration, RWD: red blood cell distribution width, PLT: platelet, MPV: mean platelet volume.


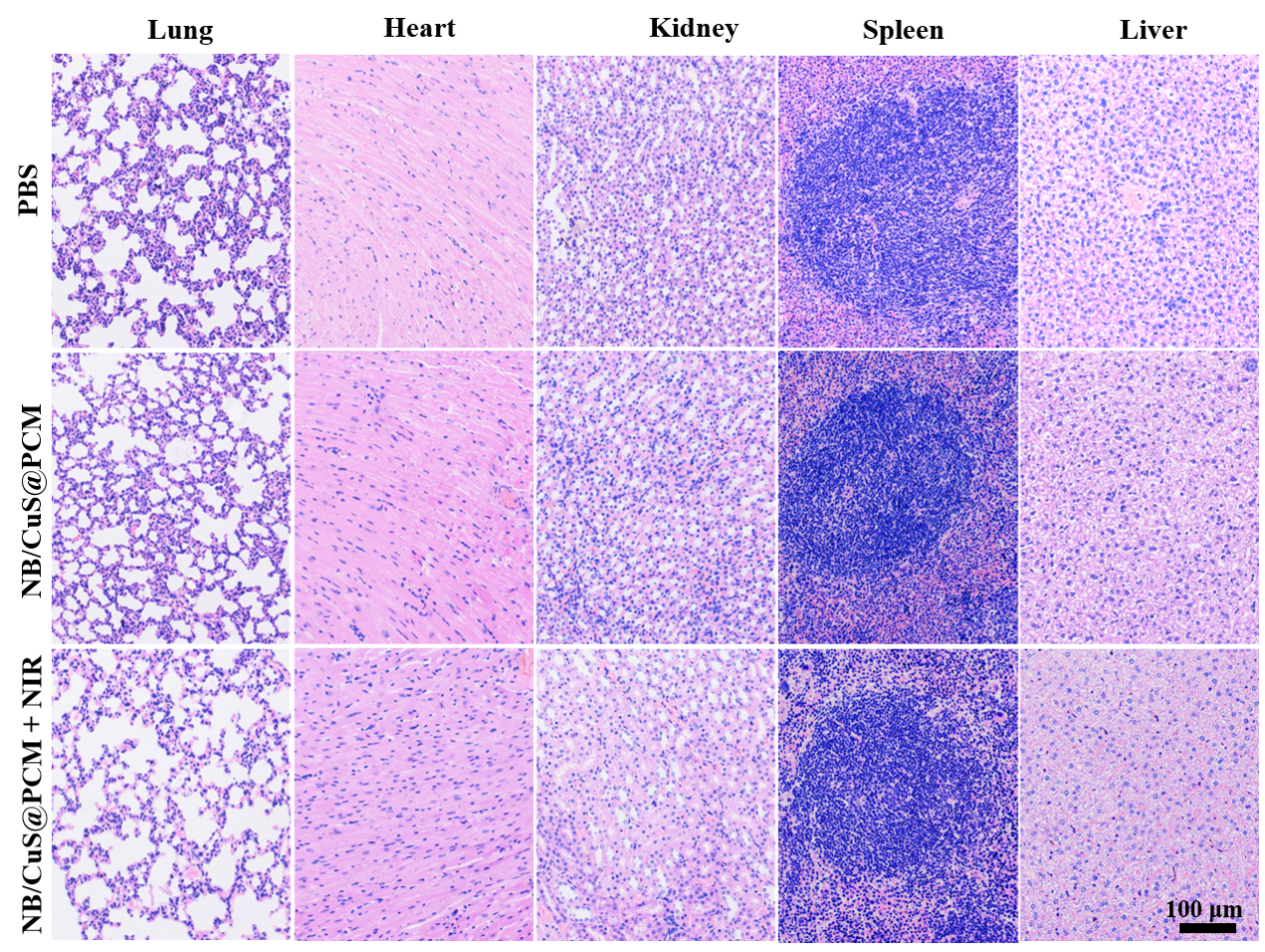


**Supplementary Figure 9.** Histopathological examination of the main organs of mice by H&E staining.
